# Draft Genome Sequence of an Extensively Drug-Resistant Salmonella enterica Serovar Typhi Strain from a Returned Traveler from Pakistan

**DOI:** 10.1128/MRA.00427-20

**Published:** 2020-07-30

**Authors:** Samantha Hao, Tess Veuthey, Saharai Caldera, Paula Hayakawa Serpa, Barbara Haller, Michelle Tan, Norma Neff, Sharline Madera, Charles Langelier

**Affiliations:** a Chan Zuckerberg Biohub, San Francisco, California, USA; b Department of Medicine, University of California, San Francisco, California, USA; c Division of Infectious Diseases, Department of Medicine, University of California, San Francisco, California, USA; d Department of Laboratory Medicine, University of California, San Francisco, California, USA; e Zuckerberg San Francisco General Hospital, San Francisco, California, USA; Indiana University, Bloomington

## Abstract

We report a draft genome sequence of extensively drug-resistant (XDR) Salmonella enterica serotype Typhi isolated from a returned traveler from Pakistan who developed sepsis. Whole-genome sequencing revealed relatedness to a previously reported outbreak in Pakistan and identified the *bla*_CTX-M-15_ and *qnrS* resistance genes.

## ANNOUNCEMENT

The emergence of extensively drug-resistant (XDR) Salmonella enterica serotype Typhi (*S.* Typhi) is a significant public concern due to the potential for severe disease with few antimicrobial treatment options (1). Whole-genome sequencing (WGS) is a valuable tool for tracking and characterizing transmission of *S.* Typhi and other pathogens (1, 2). Here, we performed WGS on an XDR *S.* Typhi isolate from a traveler who had visited Pakistan (Table 1) and developed sepsis upon return to California.

**TABLE 1:** Salmonella enterica serovar Typhi antimicrobial susceptibility testing results

Antibiotic	MIC (mg/liter)	Susceptibility
Amikacin	≤16	Susceptible
Ampicillin	>16	Resistant
Ampicillin-sulbactam	>16/18	Resistant
Azithromycin	4	Susceptible
Cefazolin	>4	Resistant
Cefepime	>16	Resistant
Cefotaxime	>32	Resistant
Ceftazidime	>16	Resistant
Ceftriaxone	>32	Resistant
Cefoxime	>16	Resistant
Ertapenem	≤0.5	Susceptible
Gentamicin	≤2	Susceptible
Imipenem	≤1	Susceptible
Levofloxacin	4	Intermediate
Meropenem	≤1	Susceptible
Piperacillin-tazobactam	32	Intermediate
Tobramycin	≤4	Susceptible
Trimethoprim-sulfamethoxazole	>2/38	Resistant

Blood cultures were performed using Bactec (BD) culture bottles and the Bactec FX instrument. Susceptibility testing was performed using the MicroScan WalkAway 96 plus instrument (Beckman Coulter). DNA extraction was carried out using the Zymo Quick-DNA fungal/bacterial kit according to the manufacturer’s instructions, followed by library preparation using the NEBNext Ultra II DNA kit. Illumina sequencing was carried out on a MiSeq instrument to yield 6,805,326 150-bp reads (2). Assembly with Unicycler v0.4.8 (3) yielded 64 contigs with an *N*_50_ value of 204,170 bp and a draft genome size of 4,757,010 bp. The GC content (52.04%) was comparable to that of other *S.* Typhi strains from Pakistan (1).

The short reads were adapter trimmed, quality controlled with fastp v0.20.0, and analyzed using the core single-nucleotide polymorphism (SNP) detection pipeline SPID v0.4.0 (https://github.com/czbiohub/Spid.jl) with a previously reported Pakistan chromosomal reference sequence for alignment (1, 4). The consensus sequence alignment was used as input to RAxML v8.2.12 to build a phylogenetic tree (5). Alignment against the plasmid reference sequence was performed with Bowtie 2 v2.4.1 (6). Mobile genetic elements were annotated with Prokka v1.14.0 (7). Draft assembly annotation was added with the NCBI Prokaryotic Genome Annotation Pipeline (8). All software was used with default settings.

**FIG 1:**
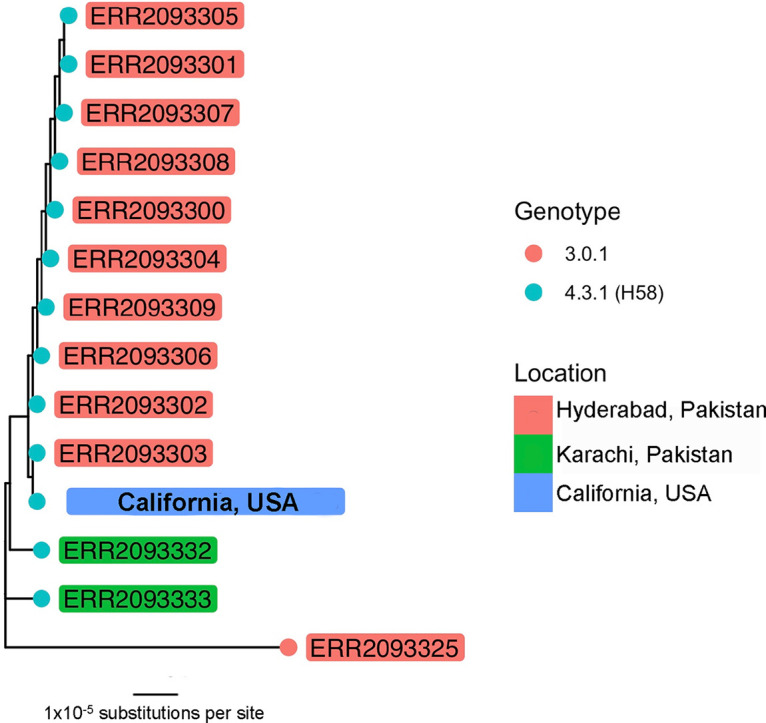
Phylogenetic tree showing the relatedness of the California XDR *S.* Typhi isolate to a subset of isolates from patients in Pakistan (1). The tree was built using RAxML with the SPID alignment as input. The label color corresponds to sample collection location. Nodes are colored by genotype according to an SNP-based typing scheme. The scale bar indicates the mean number of substitutions per site.

WGS revealed the isolate to be clonal with previously identified XDR *S.* Typhi isolates from Pakistan (Fig. 1). However, no plasmids were assembled, and alignment of the short-read data to the Pakistan reference IncY plasmid containing the *qnrS* and *bla*_CTX-M-15_ genes revealed that only partial genomic content of the plasmid could be detected. As previously reported (1), the plasmid shares several regions of homology with a chromosomally integrated antimicrobial resistance cassette, which could mediate partial integration of the plasmid into the chromosome by transposition or homologous recombination, potentially explaining this finding. Annotation of the sequences homologous to the IncY plasmid revealed both resistance genes and known mobile genetic elements, including IS*1380* family transposase IS*Ecp1*, IS*110* family transposase IS*5075*, and the Tn*3* transposon.

The draft genome sequence from this patient and the case report extends our understanding of XDR *S.* Typhi clinical presentation, pathogen evolution, and global transmission.

Analyses in this study were carried out under University of California Institutional Review Board (IRB) protocol number 17-24056.

### Data availability.

All the data are available under BioProject number PRJNA588448. The short reads are available under SRA accession number SRX7117257. The genome assembly is available under GenBank accession number GCA_009724275.1.
